# Supplementary material for: Active transport of a passive colloid in a bath of run-and-tumble particles
Source: Sci Rep. 2024 May 23;14:11844. doi: 10.1038/s41598-024-62396-2 (PMC11116446; doi:10.1038/s41598-024-62396-2)
Supplement: Supplementary file 1 — Supplementary Information 1. [file 41598_2024_62396_MOESM1_ESM.docx]

**Video legend:**

Video showing a representation simulation of the transport of a passive colloid (blue circle) in a bath of run-and-tumble microswimmers (red dots).
